# A Human iPSC-derived 3D platform using primary brain cancer cells to study drug development and personalized medicine

**DOI:** 10.1038/s41598-018-38130-0

**Published:** 2019-02-05

**Authors:** Simon Plummer, Stephanie Wallace, Graeme Ball, Roslyn Lloyd, Paula Schiapparelli, Alfredo Quiñones-Hinojosa, Thomas Hartung, David Pamies

**Affiliations:** 1 MicroMatrices Associates Ltd, Dundee, DD15JJ Scotland; 2 Dundee University Imaging Facility, School of Life Sciences, University of Dundee, Dundee, DD1 5EH Scotland; 3 PerkinElmer, 68 Elm Street, Hopkinton, Massachusetts 01748 USA; 4 Department of Neurosurgery, Mayo Clinic College of Medicine, Jacksonville, FL USA; 5 Center for Alternatives to Animal Testing (CAAT), Johns Hopkins University, 615 North Wolfe Street, Baltimore, MD 21205 USA; 6 CAAT-Europe, University of Konstanz, Konstanz, Germany; 7 Department of Physiology, University of Lausanne, Lausanne, Switzerland

## Abstract

A high throughput histology (microTMA) platform was applied for testing drugs against tumors in a novel 3D heterotypic glioblastoma brain sphere (gBS) model consisting of glioblastoma tumor cells, iPSC-derived neurons, glial cells and astrocytes grown in a spheroid. The differential responses of gBS tumors and normal neuronal cells to sustained treatments with anti-cancer drugs temozolomide (TMZ) and doxorubicin (DOX) were investigated. gBS were exposed to TMZ or DOX over a 7-day period. Untreated gBS tumors increased in size over a 4-week culture period, however, there was no increase in the number of normal neuronal cells. TMZ (100 uM) and DOX (0.3 uM) treatments caused ~30% (P~0.07) and ~80% (P < 0.001) decreases in the size of the tumors, respectively. Neither treatment altered the number of normal neuronal cells in the model. The anti-tumor effects of TMZ and DOX were mediated in part by selective induction of apoptosis. This platform provides a novel approach for screening new anti-glioblastoma agents and evaluating different treatment options for a given patient.

## Introduction

Drug development costs are high and the process is inefficient^1^. Drug companies aim to produce drugs to treat chronic and complex diseases with a high safety margin. This process involves trials with large patient sample sizes, long follow-up of patients and complex analyses^2^. The cost per drug is estimated at $1.2–1.3 billion dollars^3^, and the total development time is approximately 8 years^4^. In addition, only a small percentage of products reach the market after clinical testing, making it difficult to produce much needed new treatments for cancer patients^1^.

Preclinical drug development uses animal testing, and ~15 million animals per year are used worldwide in experimentation or to supply the biomedical industry^5^. The lack of correlation between animal models and human diseases indicates that animals are a suboptimal model to study human physiology, contributing to the high failure rate in drug development^6–8^. New approaches that rely on molecular pathways of human toxicity have been proposed to overcome drug development inefficiencies^9,10^. The development of new primary human cell culture technologies such as 3D culture, microfluidics and microfabrication in combination with human induced pluripotent stem cell (iPSC) derived models promise to generate more relevant human physiological systems for drug testing^11^. ‘Human on a chip’ systems containing several organotypic models linked together with microfluidic perfusion are promising but there are challenges to applying this approach in high throughput^12^. Spheroid models from primary human tissues offer a solution in this regard because they can be produced in large numbers with high uniformity and thus offer an opportunity for implementation of drug testing at an earlier stage in preclinical development^13^.

Performing high-throughput testing of 3D models is challenging due to difficulties associated with staining and imaging throughout the tissues caused by lack of antibody penetration and fluorescence light scatter and quenching^14,15^. To address this issue, we have developed a spheroid tissue microarray (microTMA) technology which facilitates multiplex staining and high-throughput histology analysis of spheroids^16^. The advantage of this technology is that it provides a platform for automated multiplex immunostaining of a broad spectrum of efficacy/toxicity end points and thus can be tailored for testing new therapies^17^.

Our laboratory previously reported a reproducible iPSC human-derived 3D organotypic culture, BrainSpheres (BS), that displays several characteristics of the central nervous system (CNS): BS are composed of different neuronal phenotypes, astrocytes and oligodendrocytes and have shown myelin axonal wrapping and spontaneous electrophysiological activity^18^. Moreover, they have been shown to be a reliable tool for neurotoxicology^19^. In this study, we have for the first time incorporated cells from the most devastating brain cancer (glioblastoma) from primary brain tumor tissue from our patients into the BS. This allows the study of tumor pathophysiology and drug response in a physiological environment. Glioblastoma is an aggressive brain tumor with a poor prognosis (12–14 months) due in part to its invasive nature^20,21^; hence, there is a pressing need to develop new therapies to combat this currently incurable disease. The existing therapy for glioblastoma involves surgery followed by radiation and temozolomide (TMZ) treatment^22^. To address the issue of drug resistance due to O6-alkylguanine-DNA alkyltransferase mediated DNA repair, recent clinical studies have explored more prolonged TMZ treatments on the basis that the enzyme is irreversibly inactivated during O6-alkylguanine removal and thus can be depleted by prolonged TMZ treatment^22,23^. We also chose to test doxorubicin in this context because there are several reports showing that doxorubicin is a potent anti-cancer agent in glioblastoma cell lines and *in vivo* models providing a rationale for exploring this agent clinically^24–27^. Using our microTMA technology combined with image analysis, we have been able to track the evolution and treatment of glioblastoma over time. A unique feature of this system is its ability to assess both on-target and off-target effects of drugs as our model incorporates both primary brain tumor cells from our patients and normal neuronal cells (neurons, astrocytes, glial cells).

## Results

### Characterisation of the gBS spheroid model

**Figure 1:**
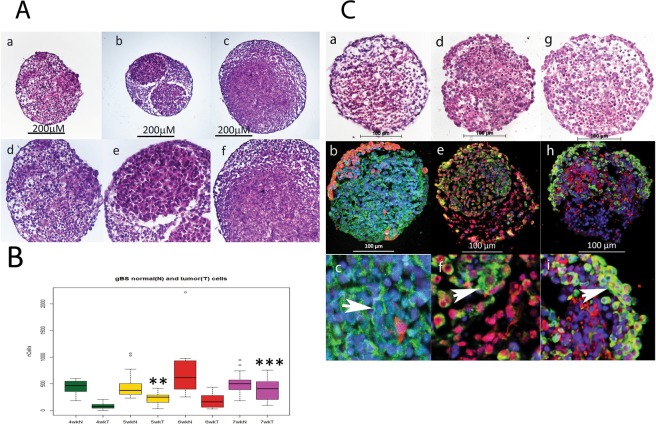
Growth of glioblastoma tumors in the gBS model. Tumour cell growth was monitored by segmenting tumour cells in the GFP channel so that we could measure tumour and normal neuronal cell numbers inside the spheroids independently. Panel (A) shows the growth of glioblastoma cells and non-tumor (normal) cells in the gBS spheroids over time (4–7 weeks). a–c H&Es (x10) show the glioblastoma cells (‘pink’ eosinophylic cells) and normal neuronal cells (lighter pink, less eosinophylic) in the gBS spheroids at different times after seeding the cultures: a - 4 weeks; b - 5 weeks; c - 7 weeks; d–f are higher powered (x25) images of the same spheroid sections shown in the top panels. Panel (B) box plots of image analysis data showing total number of glioblastoma cells and total number of normal neuronal cells in the gBS over the time course (3–7 weeks) expressed as the total numbers of cells in the spheroid section. Box plots show the median (black horizontal line) and the upper and lower quartiles (ends of the boxes). Open circles show outliers >1.5 times the interquartile range away from the upper and lower quartiles. **Significantly different from 4 wk P < 0.001, n = 20; ***significantly different from 4 wk P < 0.00001, n = 20. Panel (C) Shows representative H&E and GFP/Tuj1, GFAP/O1 and Vimentin/NF200 dual IF staining photomicrographs of untreated gBS (parallel sections): a, H&E; b GFP(red)/Tuj1(green) IF staining at low power (x20); c GFP/Tuj1 IF staining at high power (x63); d, H&E; e, GFAP (red)/O1 (green) IF staining at low power (x20); f, GFAP/O1 IF staining at high power (x63); g, H&E; h, NF200 (red)/vimentin (green) IF staining at low power (x20); i, NF200/vimentin IF staining at high power (x63). Blue staining is DAPI (nuclear counterstain).

**Figure 2:**
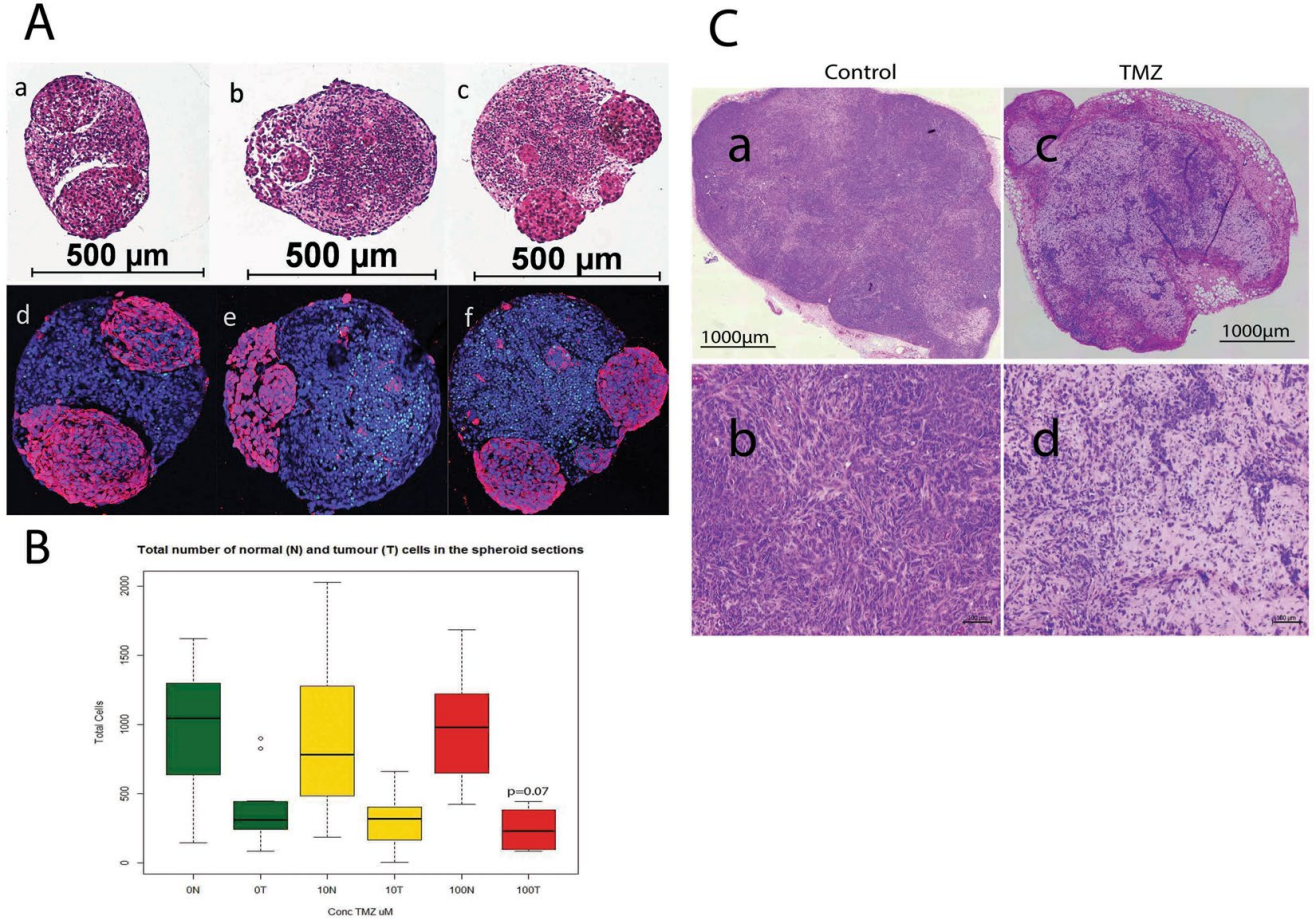
Effect of temozolomide (TMZ) treatment (10 and 100 uM) on the size of glioblastomas *in vitro* (gBS) and *in vivo* (nude mouse xenografts). Image analysis in the spheroid sections was performed by segmentation of tumour cells in the GFP channel in order to quantify their number relative to that of normal neuronal cells which were GFP negative (see materials and methods section 4.8 for details). Panel (A) a - H&E control; b - H&E 10 uM TZ; c - H&E 100 uM TMZ (glioblastomas are eosinophylic ‘pink’ cells); d–f show dual immunofluorescence (IF) stain of GFP (red) and cleaved caspase (green) with hoechst (blue) counterstain for control, 10 uM TMZ and 100 uM TZ, respectively. Panel (B) shows the total number of normal neuronal cells (N) and the total number of glioblastoma cells (T) in the spheroid sections.Box plots show the median (black horizontal line) and the upper and lower quartiles (ends of the boxes); whiskers show maximum and minimum values; open circles show outliers >1.5 times the interquartile range away from the upper and lower quartiles, n = 12. Panel (C): a, b show H&E stained sections of an untreated 965 xenograft tumor; c,d show H&E stained sections of a 965 xenograft tumor taken from a mouse treated with TMZ (100 μg/Kg)- see materials and methods section for details of the TMZ treatment(s).

We measured the number of glioblastoma cells and normal neuronal cells over a 4-week period from 3 to 7 weeks after the gBS spheroids were first formed (week 0). The number of glioblastoma cells increased in a time-dependent fashion from 3 to 7 weeks (Fig. 1A,a–f and B). By contrast, neuronal cells did not increase in number over this time course, which concurs with their postmitotic differentiation state (Fig. 1B).The histological characteristics of the gBS glioblastoma tumors were consistent with those observed in xenograft tumors with the same glioblstoma cell line in that they showed high cellularity with a spherical morphology (Figs 1A,a–f and 2C,a,b**)**.

We also found staining for the neuronal (Tuj1 and NF200), astrocyte (GFAP and vimentin) and oligodendrocyte (O1) markers consistent with the presence of these normal brain cell types in the gBS (Fig. 1C,a–i). Interestingly O1 (oligodendrocyte marker) and vimentin (astrocyte marker) were also expressed in glioblastoma tumor cells (Fig. 1C,e,f and h,i, respectively).

### Effect of Temozolomide (TMZ) treatment

gBS spheroids were exposed to TMZ (10,100 uM) over a 7-day period from the start of week 4 after spheroid formation. TMZ treatment (100 uM) caused a ~30% (p = 0.07) reduction in the total number of tumor cells (relative to control) in the gBS spheroids (Fig. 2A,a–f and B). By contrast TMZ had no effect on the total number of normal cells in the spheroids (Fig. 2B).These results are consistent with TMZ treatment *in vivo*. We tested two cycles of daily intraperitoneal administration of TMZ (100 mg/Kg, 7 days per cycle) in mouse xenograft tumors derived from the 965 glioblastoma cell line. We found that TMZ produced shrinkage of subcutaneous tumors (Fig. 2C,c,d).

### Effect of Doxorubicin (DOX) treatment

**Figure 3:**
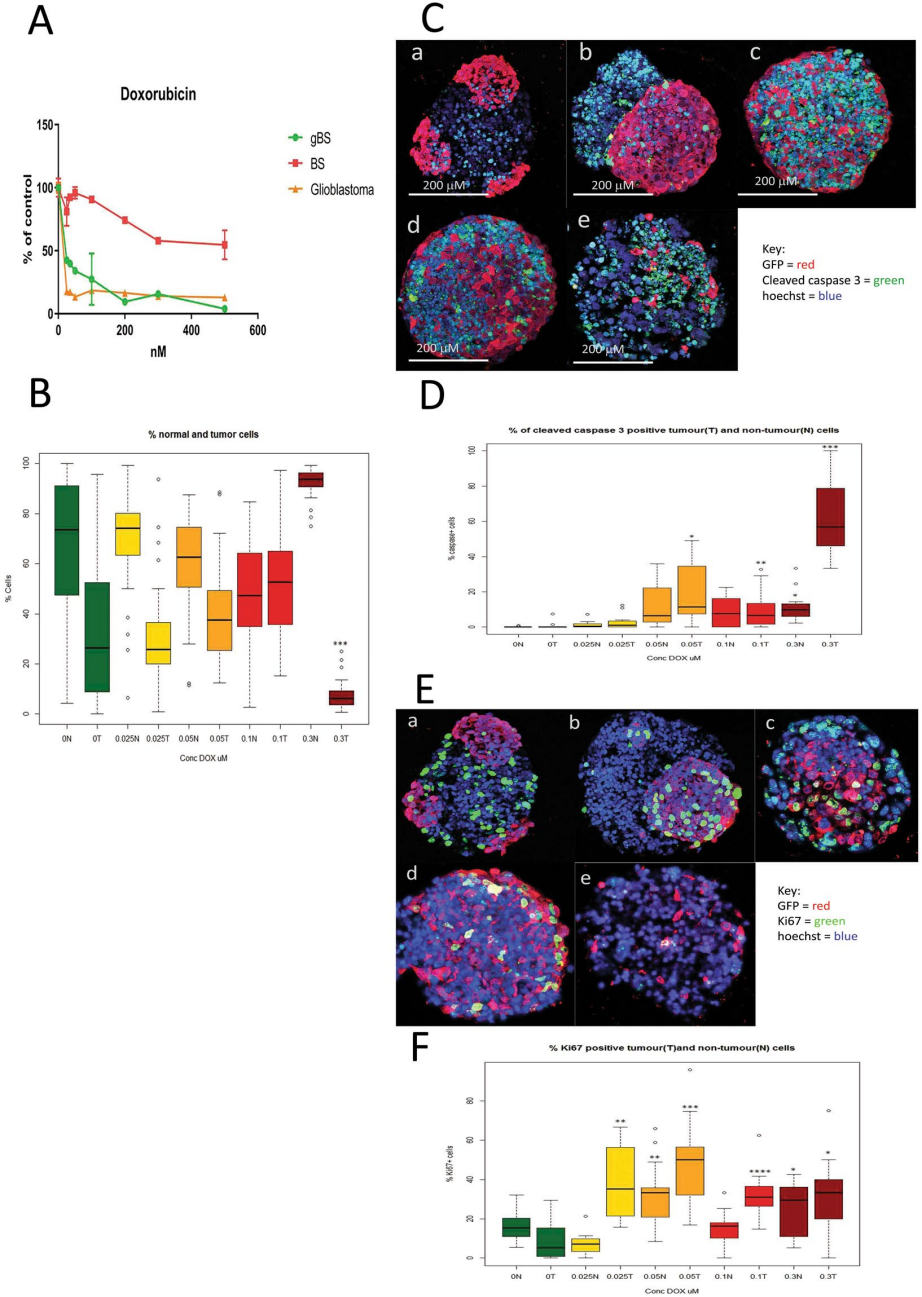
Effects of doxorubicin (DOX) treatment(s) on cell viability, tumor size and caspase or Ki67 expression in glioblastoma and non-tumor (normal) spheroid tissues. Cleaved caspase 3 and Ki67 expression in tumour cells were measured by first segmentation in the GFP channel followed by thresholding in the caspase/ki67 channel facilitating the measurement of these end-points (both expressed as percentages of caspase/Ki67 positive cells relative to total cells) differentially in tumour cells and normal neuronal cells (see materials and methods section 4.8). Panel (A) shows effects of DOX treatments (0.025–0.5 uM) on cell viability (relative to control) as measured by the resazurin assay. Results are means ± SD, n = 3. Panel (B) shows effects of DOX treatment (0.025–0.3 uM) on the total number of non-tumor (normal neuronal) cells and the total number of glioblastoma cells in the gBS spheroid sections. Values are expressed as percentages of the total numbers of cells (normal + tumour) in the spheroid sections. Panel (C) shows effects of DOX treatment (0.025–0.3 uM) on the number of cleaved caspase 3 positive glioblastoma cells and non-tumor (normal neuronal) cells in representative dual immunofluorescence (GFP/cleaved caspase 3) stained gBS sections from the microTMA: a - control; b - 0.025 uM DOX; c - 0.05 uM DOX; d - 0.1 uM DOX; e - 0.3 uM DOX. Panel (D) shows the number of cleaved caspase 3 positive tumour (glioblastoma) cells and non-tumour cells in the spheroid sections expressed as a percentage of the total cells in the spheroid section. Values are expressed as percentages of the total numbers of glioblastoma or normal neuronal cells in the spheroid sections. Panel (E) shows effects of doxorubicin (DOX) treatment (0.025–0.3 uM) on the number of Ki67 positive glioblastoma cells and neuronal cells in representative dual immunofluorescence (GFP/Ki67) stained gBS sections from the microTMA: a - control; b - 0.025 uM DOX; c - 0.05 uM DOX; d - 0.1 uM DOX; e - 0.3 uM DOX. Panel (F) shows the number of Ki67 positive tumor cells and normal cells in the spheroid sections. Values are expressed as percentages of the total numbers of cells (normal + tumour) in the spheroid sections. Box plots show the median (black horizontal line) and the upper and lower quartiles (ends of the boxes); n = 32. Open circles show outliers >1.5 times the interquartile range away from the upper and lower quartiles. ***Significantly different from control p < 0.0001 by t test, n = 32.

gBS spheroids were exposed to DOX (0.025–0.3 uM) over a 7-day period from the start of week 4 after spheroid formation. Exposure of BS and gBS to DOX (0.025–0.5 uM) caused a dose-dependent reduction in cell viability in both types of spheroids. However, the gBS were more sensitive to the cytotoxic effects of DOX compared to the BS (Fig. 3A).

Exposure to DOX (0.025–0.1 uM) had little or no effect on the size of the glioblastoma tumors, however, the 0.3 uM concentration caused a marked ~80% reduction (p < 0.001) in the size of the tumors relative to the normal neuronal cells (Fig. 3B).

### Mechanism(s) of anti-cancer activity

To assess mechanism(s) of glioblastoma cell death/reduction we measured the percentages of cleaved-caspase-3-positive and Ki67-positive glioblastoma and normal neuronal cells in the gBS spheroids. DOX treatment caused a marked (~10–60%) dose-dependent increase in the number of cleaved caspase-3-positive glioblastoma cells (Fig. 3C,a–e and D). In contrast, this treatment only caused a small (~1–10%) increase in the number of cleaved-caspase-3-positive normal neuronal cells (Fig. 3D).

DOX treatment also caused a significant (P < 0.001) increase in the number of Ki67-positive glioblastoma cells at the low doses (0.025–0.1 uM), but this returned almost to control levels at the 0.3 uM dose (Fig. 3E,a–e and F). DOX treatment also caused slight increases in the number of Ki67-positive normal neuronal cells at the 0.05 and 0.3 uM concentrations (Fig. 3F).

### Automated analysis of microTMA slides

**Figure 4:**
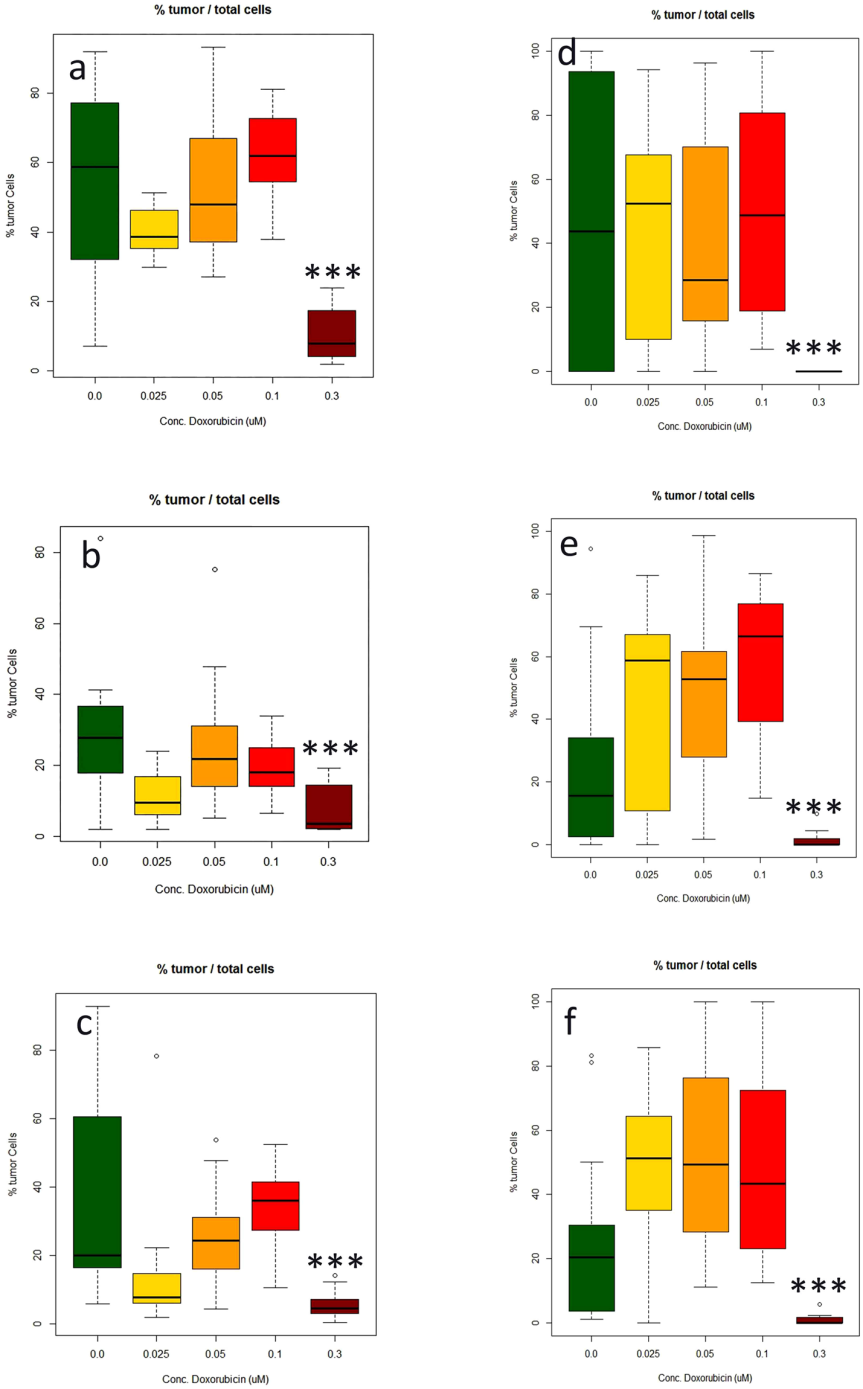
Measurement of the effect of doxorubicin (DOX) treatment (0.025–0.3 uM) on the number of glioblastoma cells made in three different microTMAsections of the same array. (**a**–**c**) Manual image acquisition (Zeiss LSM confocal); (**d**–**f)** automated image acquisition and analysis (Perkin Elmer Polaris/Inform software). Values are expressed as percentages of the total numbers of glioblastoma cells in the spheroid sections relative to the total number of glioblastoma and normal neuronal cells. ***Significantly different from control p < 0.0001 by t test, n = 16.

Tumor size and normal neuronal cell number data generated using the Polaris/Inform system was comparable to that generated from microTMA images collected manually, (Fig. 4a,d). However, the time for the image acquisition process was reduced from approximately 2 hours to approximately 5 minutes which made it feasible to scan and analyse multiple slides in a single run.

As our microTMA analysis approach was based on analysing images from single 6-micron-sections through the microTMA (96 spheroids), we validated the approach by repeating the same analysis on three different parallel sections from different levels of the same microTMA. These tests showed that regardless of the section depth, the results obtained were similar indicating that it was possible to get representative data from a single microTMA section (Fig. 4a–f).

## Discussion

Testing of anti-glioblastoma drugs in human organoid cultures is challenging due to a lack of relevant human-derived brain tumor models and difficulties associated with measuring endpoints that predict drug efficacy. Moreover, there is a lack of tools in the clinical setting to predict tumor responses and help guide the chemotherapy treatment options for a given patient. To address these issues, we have developed a human glioblastoma spheroid microphysiological system (gBS) containing normal human neuronal cells and primary human glioblastoma cells from our patients grown together in a heterotypic spheroid model. We also developed a technology for high-throughput histological analysis of spheroids and tested an existing glioblastoma therapy, i.e. TMZ, and an experimental therapy, i.e. DOX, for anti-tumor efficacy in the model.

Significant challenges with cancer drug efficacy include the variability of the response to drug treatment and the acquisition of resistance. For TMZ this is due in part to the genotype/phenotype of the tumor in regards to DNA repair enzymes^28,29^. New targets that modulate the expression of these enzymes and tumor invasion/migration have recently been discovered^30–35^. As we have shown, it is possible to grow glioblastomas in a human-relevant brain model; our approach offers a way to study anti-cancer drug response heterogeneity more rapidly than the current *in vivo* approaches^36^. By modelling the prolonged treatment of TMZ used previously in our nude mouse study in our gBS system, we have been able to demonstrate efficacy in the gBS similar to that observed *in vivo*. Given that there are publicly available libraries of glioblastoma patient-derived orthotopic xenograft (PDOX) cell lines^36–38^, we envision the creation of a more efficient discovery platform for new therapies, which ultimately offers a personalized approach by matching patients to therapies that are more likely to work clinically.

We found that TMZ and DOX treatments caused a decrease in the size of the gBS with little or no effect on the number of normal neuronal cells. Hence, our preliminary results indicate that the model can predict clinical efficacy, supporting its value in the preclinical evaluation of glioblastoma therapies. Our findings suggest that the mechanism(s) of anti-tumor action was mediated in part by selective induction of apoptosis in the glioblastoma cells relative to the normal neuronal cells.

The observation that DOX may possess anti-glioblastoma efficacy is in agreement with recent reports that showed an albumin-conjugated form of DOX (aldoxorubicin), which crosses the blood brain barrier better than DOX, was more effective than DOX in a nude mouse glioblastoma model and caused remissions of the glioblastoma in patients treated with this experimental therapy^39–41^. DOX has previously been shown to be effective at killing glioblastoma cells in a non-heterotypic glioblastoma model^42^. To our knowledge, this is the first time that DOX has been found to selectively kill glioblastoma cells in the context of a heterotypic model containing normal neuronal cells. In future studies it may be possible to model the effects of the blood brain barrier on the efficacy of anti-cancer drugs through the addition of endothelial cells to the gBS model to investigate the bioavailability of anti-glioblastoma drugs in the brain. Unlike tumors *in vivo*, the gBS spheroids lack a capillary blood supply, possibly compromising the penetration of drug into the model system. However, recent MALDI imaging mass spectrometry studies with water-soluble anti-cancer drugs have shown that there is penetration of the drug throughout tumor spheroids of 200–500 microns diameter within 6–12 hr^43,44^. Hence it seems unlikely that this would be a significant confounding factor in the gBS spheroids, which have a diameter of approximately 350microns^18^.

Other investigators have reported promising results using 3D models which recapitulate clinical scenarios better than 2D systems^32,45–47^. However, this is the first study where the effects of drug treatments have been examined in both glioblastoma and normal neuronal cells. This model is advantageous because it enables the assessment of drug effects on glioblastoma in a microenvironment more representative of the clinical situation, facilitating assessment of clinically relevant end-points such as the on-target and off-target effects of drugs and tumor invasion into normal brain tissue.It also opens up possibilities for personalized medicine with the ability to study each patient’s response to therapy in a “mini brain” model.

Our microTMA is an efficient way of multiplexing anti-tumor measurements in the same experiment. In this example, we measured the effects of the drugs on apoptosis and cell proliferation biomarkers in parallel sections. The microTMA facilitates the measurement of multiple endpoints on a single slide as, unlike whole-mount preparations, there are no limitations with regard to the use of repeated antigen retrieval cycles that are required for performing multiplexed immunofluorescence staining^48^. This enables measurement of complex drug responses involving multiple end-points at the pathway level. Ultimately this will lead to a better understanding of how a drug works and potentially provide informative biomarkers that translate to the clinic.

In conclusion, the work presented here indicates that the combination of iPSC, 3D culture and multiplexed high-throughput histology provides a novel platform for both cancer drug discovery and personalized medicine, as well as offering an alternative to animal *in vivo* studies.

## Material and Methods

### Chemicals

Doxorubicin (44583-1MG) and temozolomide (T-2577-25MG) were supplied by (Sigma). Stocks of 172 uM and 51.5 uM, respectively, were prepared in DMSO Hybri-Max (Sigma).

### Patient samples and Glioblastoma cells

Patient samples of glioma tissues were obtained at the Johns Hopkins Hospital; informed patient consent was acquired from patients and all methods were carried under the approval of the Institutional Review Board of Johns Hopkins University. All experiments were performed in accordance with relevant guidelines and regulations. Human brain tumor cell lines were derived from intraoperative tissue samples from patients treated surgically for newly diagnosed glioblastoma without prior treatment. The 965 cell line used in this study was cultured in media consisting of Dulbecco’s Modified Eagle Medium: Nutrient Mixture F-12, B27 serum free supplement (Gibco), 20 ng/mL epidermal growth factor (EGF), and 20 ng/ml fibroblast-derived growth factor (FGF) as previously reported.

### Neural Progenitor cell (NPC) production

NPCs were kindly provided by Professor Hongjun Song’s lab within our joint NIH NCATS project^18^. NPCs were derived from C1 (CRL-2097) fibroblasts purchased from ATCC. Differentiation from iPSC to NPC has been previously described^49^. Cells were grown in 175 mm^2^ poly-l-ornithine and laminin-coated flasks. NPCs were expanded using KnockOut DMEM/F12, Glutamax, EGF and bFGF) as previously described^18^. Half of the media was changed every day. Cultures were maintained at 37 °C in an atmosphere of 5% CO_2_. NPCs from passage 20 to 25 were used for this study.

### Tumor-Brain Spheres differentiation

BS were generated as previously described^18^. In order to incorporate glioblastoma cells into the BS, the protocol was slightly modified as follows: NPCs were grown (as above) in 175 mm^2^ poly-l-ornithine and laminin-coated flasks. When NPCs were at 90% confluency, 7 × 10^5^ glioblastoma cells were plated on top of the NPCs. After 24 hours the cells were detached mechanically using a cell scraper (Sarstedt). The mixture of cells was pipetted repeatedly to disaggregate cell clumps. A density of 2 × 10^6^ cells per well were plated on a non-coated 6 plate-well. Cells were grown in differentiation medium (Neurobasal^®^ electro Medium (Gibco) supplemented with 5% B-27^®^ Electrophysiology (Gibco), 1% Glutamax (Gibco), 0.01 μg/ml human recombinant GDNF (Gemini), 0.01 μg/ml human recombinant BDNF (Gemini). Cultures were kept at 37 °C in an atmosphere of 5% CO_2_ under constant gyratory shaking (88 rpm, 19 mm orbit) for up to 7 weeks, Fig. 1 Supplementary Data.

### Cell viability

BS, Glioblastoma Spheres (GS) and Glioblastoma Brain Spheres (gBS) were cultured in differentiation media for 4 weeks. After 4 weeks culture the spheroids were treated for 7 days with different concentrations of temozolomide (TMZ) (10,100 uM) or doxorubicin (DOX) (0.025, 0.05, 0.1, 0.3 and 0.5 uM). The concentration range of TMZ and DOX were chosen based on clinical data of the concentrations of drug in cerebrospinal fluid (CSF) and plasma (peak plasma concentration), respectively^50,51^. The drug or vehicle treatments were performed by mixing the drug/vehicle solution with fresh differentiation media and then changing the media. Fresh media containing drug/vehicle was added on day 1, day 3, day 5 and day 7. After exposure to the drugs cell viability was determined using the resazurin assay^52^ as follows: Resazurin (100 μl of 2 mg/ml stock) in phosphate buffered saline (PBS) was added directly to the 6-well plates (2 ml/well). The plate was incubated for 3 h at 37 °C, 5% CO_2_. Afterwards, 100 μl from each well was transferred to a 96-well plate and the fluorescence of resorufin was measured at 530 nm/590 nm (excitation/emission) in a multi-well fluorometric reader CytoFluor series 4000 (PerSeptive Biosystems, Inc). Resazurin is reduced into fluorescent resorufin (blue colour) by redox reactions only in viable cells. A one-way ANOVA test with post-hoc Bonferroni test was performed to assess statistical significance of the resazurin data.

### Construction of a spheroid tissue microarray (microTMA)

**Figure 5:**
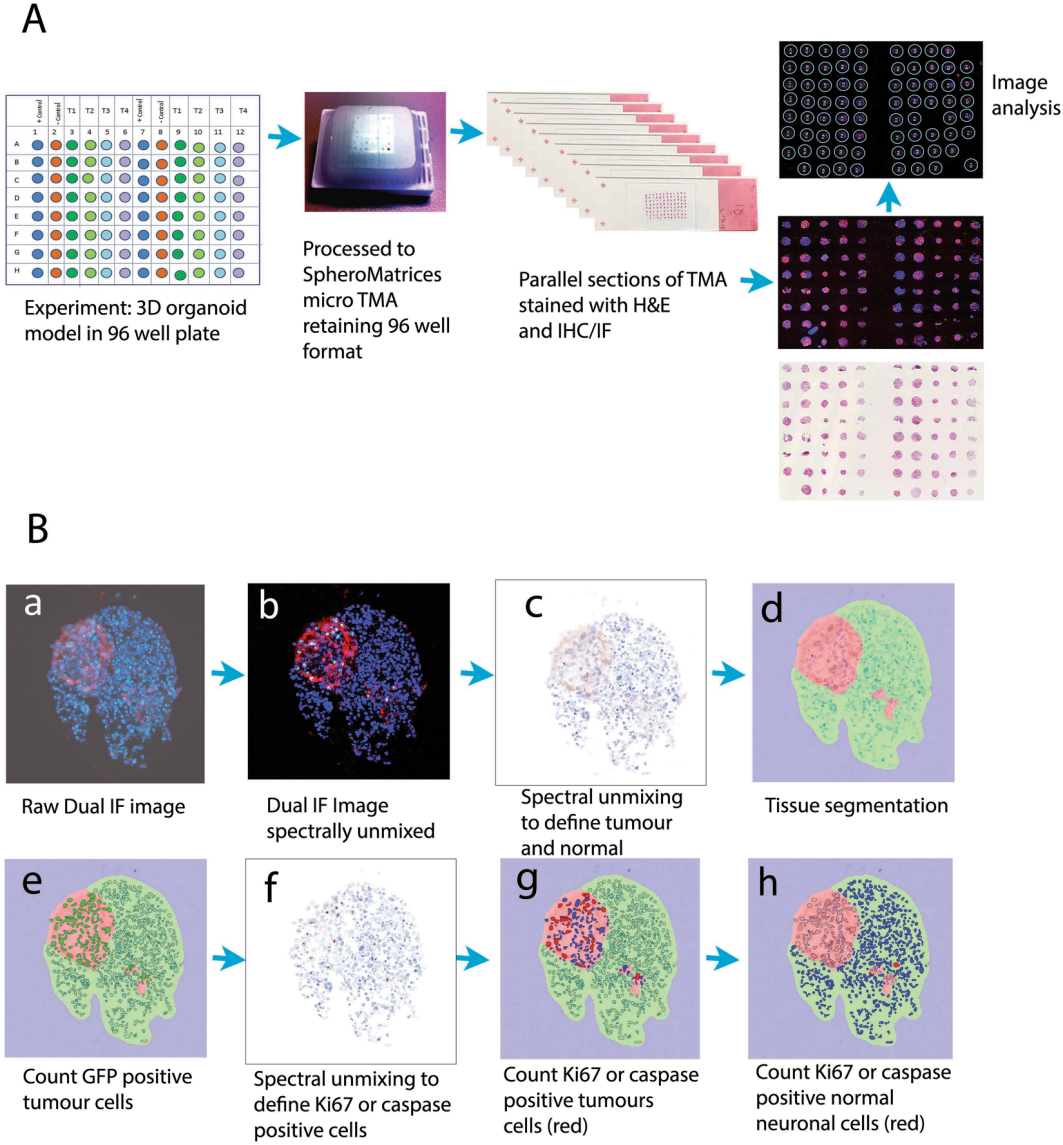
Construction and image analysis of a spheroid tissue microarray (microTMA). Fixed spheroids were embedded in a microTMA mold prior to paraffin embedding and sectioning on a microtome. TMA sections were then immunofluorescence stained and imaged using confocal scanning followed by dearraying of the spheroid images and image analysis (see material and methods section 4.8). (**A**) Schematic showing construction and processing of the microTMA for high-throughput histology and image analysis of 3D spheroid cultures. (**B**) Image analysis process to quantify tumor size and the number of cleaved caspase 3 or Ki67 positive tumor cells or normal neuronal cells. a dual immunofluorescence stained section showing GFP positive cells (red) and Ki67 positive cells (green), blue cells (DAPI) are normal (non-tumor cells); b - spectral unmixing and removal of autofluorescence; c, d - segmentation in the red channel to define tumor and normal cells/areas; e–h: segmentation in the green channel to define Ki67 or caspase positive cells, which are then counted in the tumor- and normal-cell regions.

To facilitate high-throughput histology analysis of gBS, paraformaldehyde (4%) fixed spheroids from each drug treatment were systematically organised in a microTMA using a previously published method (MicroMatrices international patent application No. PCT/GB2016/053907 publication number WO 2017/174955), (Fig. 5A**)**. Spheroids for each treatment were selected randomly from a pool of approximately 500 spheroids as illustrated in Fig. 1 Supplementary Data. Briefly, fixed spheroids were loaded into the wells of a 2% agarose mold containing 96 wells and sealed using molten 0.7% agarose. We embedded 16–32 spheroids per treatment in the microTMA mold. The agarose mold containing spheroids was dehydrated for a minimum of 12 hr in 70% ethanol and then the microTMA mold was processed to paraffin wax in a tissue processor (Thermo Citadel 1000). Following wax embedding the microTMA block was sectioned (6 uM sections) using a microtome (Reichert Jung) onto glass microscope slides (VWR Superfrost Plus).

### High-throughput histology and immunostaining analysis of gliobastomaBrainSpheres (gBS)

MicroTMA slides were dewaxed in Histoclear and then heat induced epitope retrieval (HIER) was performed using citrate buffer solution pH6 (Vector labs).Parallel sections were stained in haematoxylin and eosin or by immunostaining. Dual immunofluorescence (IF) staining using anti-green fluorescent protein (GFP) together with anti-Ki67, anti-cleaved-caspase-3, anti-beta Tubulin III (Tuj1)-antibodies was performed as follows: microTMA slides were dewaxed (Histoclear), dehydrated through graded ethanols (70%-100%) and subjected to HIER in pH 6.0 citrate buffer for 20 minutes at 121 °C in an antigen retriever (Prestige Medical). After HIER, the slides were washed in distilled water, and mounted in PBS on a Shandon disposable immunostaining chamber according to the manufacturer’s instructions. Blocking buffer (1% BSA (Sigma A7906-100G), 0.2% Triton X100 (VWR #3063324), 3% normal goat serum (Sigma G9023-10 ml) in PBS) was added to the chamber and the slides incubated for 2 hours at RT. Anti-GFP (Abcam #13770), anti-Ki67 (Abcam # 15580), anti-cleaved caspase 3 (Cell signalling #9661S) or anti-Tuj1 (Abcam #78078) primary antibodies at 1:500, 1:1000, 1:100 or 1:2000 dilutions **(**PBS/0.1% BSA, 0.5% normal goat serum, 0.2% Triton), respectively, of the stock including a primary antibody negative control (wash buffer alone) were then added to the chambers and the slides incubated at RT for 1 hr. Slides were washed in wash buffer (0.1% BSA, 0.2% Triton, 0.5% normal goat serum in PBS). A mixture of secondary antibodies, goat anti-rabbit Alexa fluor 488 (Life Technologies # A11034) and goat anti-chicken Alexa fluor 568 (Life Technologies # A11031) diluted 1:500 in wash buffer and Hoechst 33342 (Sigma #B2261) (1 mg/ml) diluted 1:1000 in wash buffer, was added to the chamber and the slides incubated for 2 hours at RT. The slides were then washed and mounted using antifade mountant (Vectashield, Vector Laboratories # H-1000). Dual IF-stained slides were imaged using a Zeiss 710 LSM confocal microscope.

### Image analysis

A custom Image J algorithm automatically processed each dual IF stained spheroid image across the microTMA to calculate (1) the total number of tumor cells (GFP-positive) as a percentage of the total number of tumor and non-tumor (neuronal) cells in each spheroid section; (2) the total number of Ki67 or caspase 3 positive tumor cells as a percentage of total tumor cells and (3) the total number of Ki67- or caspase-3-positive neuronal cells as a percentage of the total number of neuronal cells. The algorithm works by viewing the pixel intensities (grey scale) for each fluorescence channel (e.g. red, green or blue) in sequence and identifying cells with pixel intensities above threshold. The location (coordinates) of the cells is first identified in the blue channel (Hoechst 33342) in a blurred image (Difference of Gaussian), identifying the centre point of each nucleus. The algorithm then classifies/segments the cells as either tumor (above threshold red channel) or non-tumor (below threshold red channel) and subsequently Ki67^+^ or cleaved-caspase-3^+^ (above threshold green channel) or Ki67^−^ or cleaved-caspase-3^−^ (below threshold green channel). The algorithm measures average intensity about the centre of the nucleus, within the radius of the cell area (~10 microns). A minimum of 12 spheroid sections per treatment were analysed and in most cases 32 spheroid sections per treatment were analysed. We also tested whether or not analysing a single microTMA slide would generate representative data for the whole array. We analysed sections from the same microTMA block at different levels in the gBS spheroids and found that the data were comparable regardless of the microTMA section analysed indicating that the analysis of a single microTMA slide/section generates data that is representative of the whole microTMA (see results section Fig. 4).

### Automated Scanning and Multispectral Image analysis of microTMA slides

As the manual image acquisition process on the Zeiss LSM 710 confocal (above) was laborious we explored the possibility of automating the process using a PerkinElmer Vectra Polaris scanner (Vectra Polaris software version 1.0.6). A whole slide scan was performed at 20x, then a modified version of the ‘TMA’ function in Phenochart software (version 1.0.8 plus additional code) was used to find smaller spots than standard and assign coordinates to each spot on the microTMA.These were then imaged multispectrally at 20x to enable auto-fluorescence removal and un-mixing of fluorophores for score/quantitation^48^. The images were analysed in Inform image analysis software (version 2.4.1) to quantify tumor size and the number of cleaved caspase 3 positive or Ki67 positive tumor cells and normal neuronal cells in parallel sections of the microTMA, (Fig. 5B).

## Supplementary information

Supplementary info Figure 1
