# Supplementary material for: A Human iPSC-derived 3D platform using primary brain cancer cells to study drug development and personalized medicine
Source: Sci Rep. 2019 Feb 5;9:1407. doi: 10.1038/s41598-018-38130-0 (PMC6363784; doi:10.1038/s41598-018-38130-0)
Supplement: Supplementary file 1 — Supplementary info Figure 1 [file 41598_2018_38130_MOESM1_ESM.docx]

A Human iPSC-derived 3D platform using primary brain cancer cells to study drug development and personalized medicine

## **Authors**

*Simon Plummer^1*^, Stephanie Wallace^1^, Graeme Ball^2^, Roslyn Lloyd^3^, Paula Schiapparelli^4^, Alfredo Quiñones-Hinojosa^4^, Thomas Hartung^5,6^, David Pamies^5,7^*.


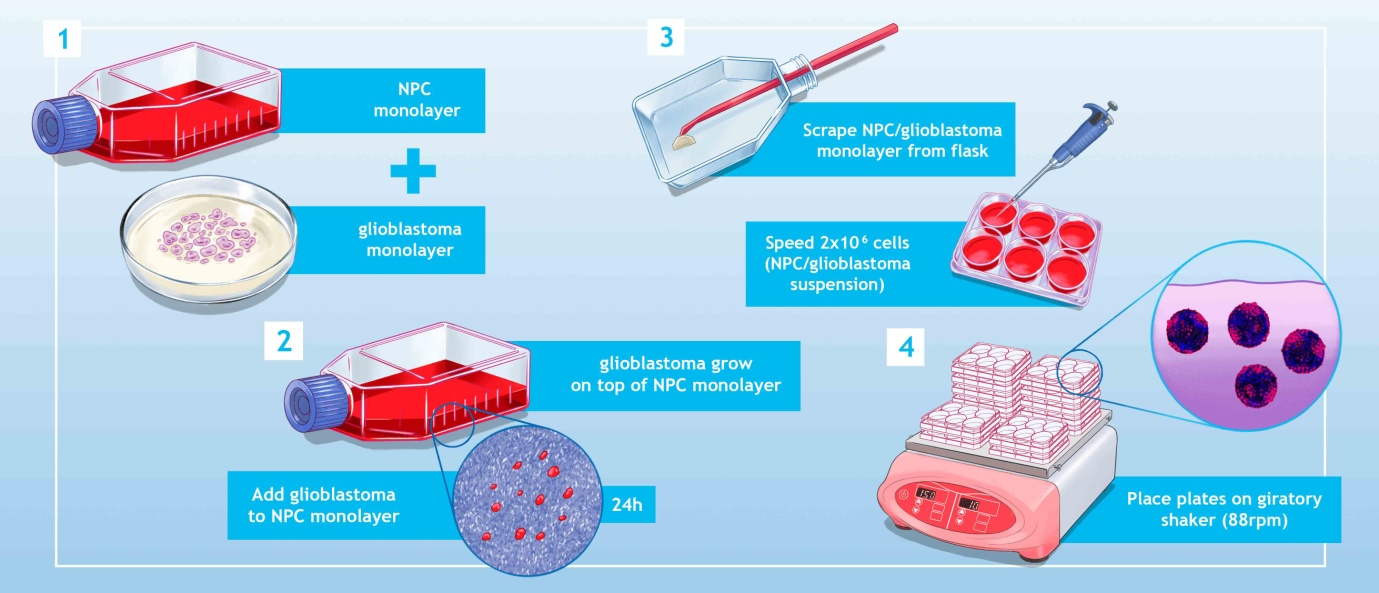


Figure 1. Supplementary data: Schematic showing the process for making the gBS. Briefly, neural progenitor cells (NPCs) and glioblastoma cells were cultured separately to form monolayers and then the glioblastoma cells were scraped off, dissociated by pipetting and seeded on top of the NPC monolayers. Once the glioblastoma cells have attached to the NPCs, the flask was scraped using a rubber policeman and the cells were dissociated by pipetting and seeded into 6-well plates. The cells were then incubated (4-7 weeks) in a CO_2_ incubator on a giratory shaker until the gBS spheroids (~500 per well) were formed. Following drug treatment(s), spheroids for microTMA analysis were selected randomly from each well.
